# Social ecosystems of trafficked conflict-related sexual violence survivors and their children: navigating and coping with intersecting stigma and violence in Nigeria and Iraq

**DOI:** 10.1186/s12939-026-02828-9

**Published:** 2026-03-31

**Authors:** Dashakti Reddy, Marguerite Daniel, Myriam Denov

**Affiliations:** 1 Department of Health Promotion and Development, University of Bergen, Alrek Helseklynge, Årstadveien 17, Bergen, Norway; 2 School of Social Work, McGill University, 550 Sherbrooke Ouest, Montreal, QC H3A 1B9 Canada

**Keywords:** Children born of war, Conflict, CRSV, Social ecological systems, Iraq, Nigeria, Stigma, Wellbeing

## Abstract

**Background:**

We explore the intersectional risk of stigma and violence faced by women and girls trafficked by armed groups, and their ‘children born of war’ (CBOW) in Nigeria and Iraq. Failing to address intersectional violence, stigma and discrimination has severe implications for women’s and children’s health, wellbeing, intergenerational trauma, sustained reintegration, and Women, Youth, Peace and Security Agendas, Security Council Resolutions 1325/2467 and Sustainable Development Goals (SGDs).

**Methods:**

Thirty-two in-depth interviews (IDIs) and eight focus group discussions (FGDs) were conducted with conflict-related sexual violence (CRSV) survivors, community, faith leaders, service providers and government stakeholders to understand experiences and perceptions of stigma, coping and reintegration challenges.

**Results:**

Findings show that experiences of mothers and their children are complex and interrelated, and that they can experience stigma at every socio-ecological level. Stigma contributed to rejection and abandonment and in some cases return to the armed groups. Stigma was often linked to the perceived affiliation with the armed groups and children’s paternity, but also to atypical religious related and aggressive behaviours. In Nigeria, initial family acceptance could erode due to community stigma, leading to eventual rejection. Stigma negatively impacted wellbeing, sustained acceptance and access to services. Yet, survivors utilised key resilience resources to cope. Protective factors included support by female relatives and faith leaders, and relocation.

**Conclusions:**

Stigma experienced by CRSV survivors mirrored stigma experienced by gender-based violence (GBV) survivors’, but they also faced new forms of conflict-related stigma, emphasising the need to address underlying gendered norms and new conflict-related stigma dynamics. Findings have important implications in understanding how community-based stigma can influence family outcomes, which is far less known. It also gives insight into areas to strengthen for practitioners working with this population, including for social workers. We contribute to the literature by focusing on two conflict-affected contexts where limited research on these topics exist.

**Supplementary Information:**

The online version contains supplementary material available at 10.1186/s12939-026-02828-9.

## Background

Stigma can cause invidious harm in any context, not least to conflict-related sexual violence (CRSV) survivors and their children. As defined by Goffman [1], stigma is an ‘*attribute that is deeply discrediting’* that can cause shame. Attributes can include physical, mental or social characteristics deemed negative, or behaviours that transgress socially accepted norms, resulting in an individual or group being discriminated against or excluded [2]. How stigma presents in a given context is complex and nuanced, but it can result in long-term and severe intergenerational consequences if left unaddressed [3]. Stigma can lead to feelings of shame and social isolation which can discourage help-seeking behaviour and limit access to essential services [3]. In turn, this can negatively impact physical and mental health and wellbeing, increasing vulnerability which can lead to harmful coping mechanisms. This can especially be the case for women and girls in conflict settings who have been trafficked, subjected to CRSV and as a consequence conceive ‘children born of war’ (CBOW), the focus of this paper. The intergenerational impact and legacy of stigma experienced by mothers and their children individually and as a unit cannot be underemphasised. A systematic qualitative review of CRSV consequences on survivors found that stigma was one of the most negative and persisting consequences, and ‘*living with children born after sexual violence related pregnancies’* (SVRP) was a key theme [4]. Stigma related to gender-based violence (GBV), including as a barrier to healthcare is well documented in high-, middle- and low-income countries [5]. For decades, research on CRSV and research in conflict settings remained limited [6]. However, a recent review reveals that CRSV research has significantly increased in the last 15 years, expanding nuanced insight into the consequences, dynamics, intersectional approaches, and on understudied survivor groups [6]. Research on the experiences and service needs of survivors of trafficking is more available [7]. Research on CRSV stigma [8–11] and research on CBOW as a distinct group in addition to their experiences of stigma is also growing [12–14]. Further, the intersection of violence against women and violence against children is also increasingly recognised [15]. However, there is little research on *stigma* at the intersection between CRSV survivors and the children they conceive as a result, particularly in Nigeria and Iraq [16–19]. In this paper, we explore survivors’ and their children’s individual and intersecting experiences of stigma from different perspectives across their socio-ecological environments by answering this question: ‘how do formerly trafficked CRSV survivors navigate and cope with stigma directed towards themselves and their CBOW during reintegration?’ A key strength of the study is that we integrate multiple voices and perspectives across survivors’ social ecologies, which is particularly pertinent to explore in how stigma interacts across different levels.

## Terminology

Overall, we use the term gender-based violence, and where relevant conflict-related sexual violence as a form of GBV, that occurs specifically in conflict or post-conflict settings [20]. This research focuses on a subgroup of female CRSV survivors: those trafficked by armed groups who conceived CBOW as a result of CRSV and returned to their communities. However, we also present the situation of GBV survivors (non-trafficked survivors). When referring to the social ecological model (SEM) of Murphy et al. [21] we use the term ‘*conflict-related violence against women and girls’* (CRVAWG) to align with their framework. Children conceived through CRSV are: *‘persons of any age conceived as a result of violent, coercive, or exploitative sexual relations in conflict zones*,’ [22] and who usually have a mother from a local community and a father from a peacekeeping force or foreign army [23]. In this paper, we specifically refer to ‘children born of war’, who are born to mothers trafficked and held in captivity, and fathers from armed groups [12]. In Iraq, we focus on the Yezidi community, where fathers were not from the Yezidi ethnic community. In Nigeria, fathers could be from the same community and same ethnic group, which we highlight as a result of the associated stigma.

## Contexts

While Nigeria and Iraq have distinct histories and demographic profiles, they share a number of factors including sitting across key geopolitical regions. Borno State in Nigeria borders the Lake Chad Basin and Sambisa forest, while Nineveh Governorate in Iraq includes Sinjar, home to the Yezidi community, and the Kurdistan Region of Iraq, and shares borders with Türkiye and Syria [24, 25]. Both have experienced prolonged internal and regional conflicts with severe consequences to human life and wellbeing. Terrorist groups and the mass trafficking of women and girls into captivity, forced marriage, religious conversion and CRSV resulting in children have also been present. Insurgencies in both countries escalated in 2009 and 2014, by Boko Haram in Nigeria and Daesh/Islamic State (IS) in Iraq, causing mass displacement [24, 25]. Both contexts also have diverse ethnic and religious populations with a largely Muslim majority. While all communities have been impacted, minority ethnic and religious groups have been particularly targeted by the armed groups [24, 25]. For example, in Iraq, Arab and minority Shabak, Turkmen and Christian communities were all impacted by the war. However, the Yezidi community, who have historically faced persecution and attack by different groups were particularly targeted [24]. In Nigeria, armed groups have indiscriminately attacked schools, churches and mosques, the later to provoke religious tension [25]. Given a key aspect to their ideology is against western education and to institute Islamic Law, conversion of non-Islamic religions has been a part of their operations [25]. Further, stakeholders have worked closely to support returnees’ reintegration [26]. However, on-going insecurity continues to challenge these efforts and impact all communities [24, 27, 28]. Despite contextual differences, comparing predictive factors for abuse, drivers of stigma, coping mechanisms, and service responses across both contexts offers valuable cross-context learning and implications for policy and programme funding decisions.

## Theoretical frameworks

A critical aspect of stigma is that it is relational and rooted in interactions between people and groups. We therefore apply two aligned Social Ecological Models, as it provides a comprehensive framework to understand how multiple layers of influence shape individual experiences, potential risk to violence and health and wellbeing outcomes. We use the SEM to explore, 1) the intersection of CRSV survivors’ and their children’s individual, collective and intergenerational experiences, 2) how community-based stigma influences other SEM levels, and 3) to identify the resilience resources survivors use to cope with stigma, compared across two contexts. Heise’s [29] SEM has been a foundational framework to understand women’s risk factors to violence. The holistic structure helps to understand how risk factors interact across different social ecologies, emphasising that violence is rarely caused by a single factor but by multiple interrelated aspects. Notably, it attempts to outline predictive factors of abuse at each level. Building on Heise’s work, Murphy et al. [21] adapted the theory and CRVAWG framework to specifically account for the unique dynamics found in conflict and post-conflict contexts. Their six layered model comprises overlapping *individual, interpersonal, community, institutional, societal and global levels*. Further, Shevell and Denov [30] reimagine a SEM to look at resilience across space and time, through an integrated intergenerational approach that focuses on the community, societal and global levels. Concepts of resilience have largely focused on an individual’s ability to ‘bounce back’ from a stressor, using strengths based coping mechanisms. However, Shevell and Denov [30] emphasise the need to consider resilience across all layers of the SEM, not just the individual. This aligns with Murphy’s et al. [21] whole system approach to understanding intersecting risks to CRVAWG. We use the CRVAWG SEM to explain experiences of stigma at the individual, interpersonal/family, community and institutional levels, outlining risk and protective factors. We draw on Shevell and Denov’s [30] model to specifically look at the impacts across time and at an intergenerational level.

## Literature

Stigma can elicit far-reaching and intergenerational consequences that cross the boundaries of time, as shown in places like in Bosnia and Rwanda, where thirty-years post-conflict survivors and CBOW continue to experience a high sense of fear and anxiety due to stigma and ostracization [20, 30]. We summarise below key sources and consequences of individual and intersectional stigma at different SEM levels.

At an individual level, survivors in the Democratic Republic of Congo (DRC) [31] and those trafficked in Nigeria [32], have reported experiencing high levels of stigmatisation, isolation, rejection, discrimination and abandonment due to sexual violence. Some have described that the social stigma from rape can be as traumatic as the attack itself [33]. Many communities often blame survivors for the attack [34], which can prevent reporting the pregnancy, the birth or the child due to associated protection risks [35]. A study in the DRC found that when women were blamed for rape, there was a stronger association to stigma [36]. Feelings of shame and humiliation can be reinforced by family and community rejection and increase survivors’ risk to complex psychological and psychosocial outcomes [31]. Outcomes can include depression, suicidal ideation, intrusive memories, isolation and withdrawal, flashbacks, and issues with identity and sleep [37]. Other consequences include negative socioeconomic factors and abandonment by spouses [38].

At the family and community levels, community-based stigma can provoke one of the strongest influences on survivors and CBOW. It can impact the mother’s ability to parent [39], and promote self-isolation which undermines community relations [40]. The fear of being stigmatised can also act as a barrier to accessing services [31]. For example, survivors in the DRC reported worsened family relations, increased verbal abuse by household members, diminished household and community status, and barriers to accessing health information and services due to fear of stigma [31, 33]. In Uganda, research revealed how family members’ own experiences of stigma, secondary stress and caring for CBOW negatively impacted their relationships with returned mothers [41]. The majority of mothers do not reject their children. However, in some contexts, families may accept survivors back but not CBOW, which occurred with the Yezidi community in Iraq [42].

Intersections of stigma and violence and the association between a mother and child’s wellbeing have been documented, including in a few cases for women with SVRPs in conflict settings [33, 43, 44]. In the DRC, one study found that the chance of family and community rejection for women with SVRPs was more than twice that of those who did not get pregnant [33]. Another study found that having a child born from rape significantly worsened survivors’ isolation in comparison with those who did not have a child [45]. Mother–child relationships in this situation are complex, they can shift and can intimately be influenced by a number of issues including stigma [14, 17, 43]. For CBOW, maternal symptoms of post-traumatic stress, stigma and isolation can affect mother-child interaction and thereby child development, recovery and reintegration [43, 46]. For survivors, CBOW can be perceived as obstacles to reintegration and raise concerns over loss of identity such as in Rwanda [44].

CBOW face particularly unique experiences of stigma, including through the global phenomena of shaming them with names such as ‘devil’s children’ in Rwanda, ‘children of shame’ in Kosovo, ‘rebel pikins’ in Sierra Leone and ‘Isis babies’ in Iraq and Syria [12, 14, 22]. Perspectives from children have shown that they recognised the challenging relationship with their mothers [13], experienced both abuse and affection, and could experience stress and fear due to role reversal and responsibilities when mothers were unable to care for themselves [47]. For example, Bosnian girls conceived of CRSV expressed weak social bonds with their communities and defined their position as ‘scapegoats’ [47]. Based on their experiences of abuse, social exclusion and stigmatisation, they believed they deserved to be hated and harassed based on having paternal ‘Serbian blood’ [47]. Yet shared trauma and shared adversity have also been shown to strengthen mother-child bonds and provide a sense of purpose and maternal duty [39].

## Methods

### Study design and setting

A qualitative approach facilitated the exploration of survivors’ lived experiences and stakeholders’ perspectives across all social ecologies in North-East Nigeria (Borno) and North-West Iraq (Nineveh). Locations were chosen due to the similar nature of CRSV and trafficking for forced marriage and conversion to another religion by armed groups. Site selection also included availability of GBV services to facilitate referrals. Insecurity and COVID-19 led to some delays and prevented some data collection but was mitigated for by extending the data collection timeline and adhering to security protocols and distancing restrictions.

### Ethics

Given the vulnerability of the population in this study, we address essential ethics early on in the methods section. The safety of participants and researchers was prioritised throughout the project through adherence to international and national guidelines. Ethics principles and participant safety and confidentiality were given precedence throughout the research to mitigate risk and distress to participants. Ethics approvals were acquired via the Norwegian Regional Committee for Medical and Health Research Ethics (REK) 322537, the Nigerian Ministry of Health: Health Research Ethics Committee 051/2021 and Nineveh Governorate – JCMC (2020/00777070) and the Ministry of Interior. Safety for survivors was prioritised and involved utilising already trained GBV caseworkers as interpreters with valuable contextual insight on risk. Interpreter de-briefings also facilitated wellbeing check-ins and clarification of some responses. Acute attention was paid to the consent process to ensure voluntary participation and the explicit right to pause or withdraw was understood. Survivors were provided the option to interview with a support person of their choice. Two survivors chose to interview together. Limits to confidentiality were emphasised during FGDs and participants were asked not to name others. Precautions were taken to ensure strict data protection, including by utilising systems for risk and compliance and processing sensitive data, and encrypted storage of data. Photographs were taken, with permission, only of the output of the FGD exercise.

### Participants

Participants at all SEM levels were interviewed to explore their experiences and perspectives. Interviews included formerly trafficked CRSV survivors forcibly married to armed groups (2 Iraq (IQ), 2 Nigeria (NI)), children associated with armed forces and armed groups (1 NI), displaced community members (4 FGDs NI), community and faith leaders (1 IQ, 5 NI), government workers (2 IQ, 4 NI), service providers (7 IQ, 8 NI) and GBV caseworkers directly supporting survivors and their children (1 FGD IQ, 3 FGDs NI). Participants were approached through the first author’s networks, after which snowballing methods were used to invite others. Inclusion and exclusion criteria were developed which followed a clear recruitment strategy to mitigate risks to participants, particularly for survivors. Exclusion criteria included persons under the age of 16 years old and persons without the ability to provide informed consent. All but two participants were nationals of the respective country.

### Data collection and analysis

Thirty-two semi-structured IDIs (to capture personal and sensitive experiences) and eight FGDs (exploring social norms) comprising 10–15 participants each were conducted between October 2021 and October 2023. Interviewers observed participants for non-verbal cues to identify unease and provide the opportunity to stop or pause the interview, which occurred in one case. IDIs and FGDs lasted 45–60 minutes, were held in private locations to maximise confidentiality and were conducted by the first author in English. Interpretation from Arabic/Kurdish languages in Iraq and Hausa/Kanuri languages in Nigeria were required for twelve IDIs/FGDs. Community members were informed by service providers about the topic of the FGDs and invited to participate. IDI/FGD guides were pre-reviewed with GBV caseworkers for cultural sensitivity. FGDs involved a free-listing exercise to draw out prevalent GBV types, followed by a vignette exercise (short hypothetical story that simulates real events followed by probing questions). GBV caseworkers acted as interpreters and were briefed beforehand to ensure direct translation. With permission, interviews were audio recorded and deleted after transcription. We used Thematic Network Analysis [48] to support an iterative and inductive coding process. All text was coded and clustered into basic, organising and global themes, providing a basis to identify patterns and analyse implications of the data, with the support of NVivo software. Two transcripts were separately coded by two authors to develop the coding framework to reduce interpretation bias and improve analysis.

## Results

Findings are outlined in four key themes; 1) stigma related to GBV that influence CRSV survivors’ experiences; 2) stigma experienced by trafficked children and CBOW; 3) the experience of stigma by trafficked CRSV survivors; and 4) factors amplifying consequences of stigma and mother-child wellbeing. We present survivors’ experiences, as told by them and perspectives of stakeholders working directly with them. Notably, more data was collected in Nigeria than Iraq. To account for this, we first frame this section by findings that were shared between the two contexts, followed by data from Nigeria and then what was different or similar in Iraq. Participant quote codes are: CRSV survivor (SV), service provider (SP), government (Govt), Yezidi Religious Leader (YRL), Muslim Religious Leader (MRL), male leader (ML), woman leader (WL), women’s FGD (WFGD), girl’s FGD (GFGD), caseworker FGD (CWFGD), Iraq (IQ), Nigeria (NI).

### Theme 1: Stigma related to GBV that influence CRSV survivors’ experiences

Stigma was associated with GBV and CRSV in both contexts and had long-term implications for survivors. Stigma was deeply rooted in existing patriarchal social, gendered and cultural norms, particularly those that tied family honour to women and girls’ virginity, behaviour and marriageability. When girls were perceived to violate norms, even if as a result of sexual violence, they were considered ‘spoiled’ (Nigeria) and ‘unhonourable’ (Iraq). Several participants in Nigeria noted that female GBV survivors were often victim-blamed and stigmatised, resulting in severe consequences such as having to leave school, prompting relocation or being forced to marry the perpetrator. Further, more stigmatisation was reported in cases where sexual violence resulted in pregnancy, linked to childbirth outside marriage and the perceived loss of honour. These same norms affected trafficked CRSV survivors. A Nigerian government worker explained the situation of returned survivors and the dynamics of returning in childhood with a CBOW: *A child with a child is not an easy case to handle. Normally we don’t even want to reunify them because they will face a lot of stigmatisation* (Govt-NI). Comparatively, in Iraq, while stigma related to premarital pregnancy was present for GBV survivors, this was not specifically raised as an issue for CRSV survivors. This was most likely influenced by the Yezidi religious leaders’ support resulting in families seeing survivors as heroines according to one service provider: *There is respect for the mother because she was in captivity, so, many community will see her as a hero* (SP-IQ).

Further, findings in Nigeria, not illustrated in Iraq, revealed that, in addition to the stigma related to the sexual violence, survivors could also face a ‘double’ stigma in three distinct scenarios, each with implications for their marriageability and reintegration as explained by several service providers. These included: 1) sexual violence and being unwed with a child, 2) sexual violence and returning *without* a child and, 3) the consequences of sexual violence, particularly related to fistula. The first relates to norms and stigma around premarital childbirth: *The ones that come out with children will have more difficulties in getting remarried … I’ve not seen one that has got married with a child* (SP-NI). The second involves stigma associated with perceived infertility: *People will be like … ‘it’s very difficult for you to go to that place and not come back without a child. It means that you have some kind of issue, you cannot give birth … you’re barren’* (SP-NI). The third concerns rejection resulting from physical health consequences of CRSV: *She was repeatedly raped and never got pregnant once … she developed VVF [vesicovaginal fistula] … [the armed group] started saying that she smells, you know, she’s dirty. So, they decided to push her out* (SP-NI).

Sensitisation efforts by service providers acted as a strong protective factor that reportedly lowered the level of perceived blame against CRSV survivors across both contexts. In Nigeria, some participants believed the increased awareness helped change community mindsets towards survivors. This was particularly evident in interviews with religious leaders who held strong views about GBV survivors and attributed blame to them based on factors such as their clothing. Comparatively, they believed it was not CRSV survivors’ ‘fault’. Similarly, in Iraq service providers noted the importance of sensitisation but also the significant impact of international pressure and the Yezidi High Council Religious Decree, an indispensable protective factor. One service provider explained: *There was a lot of programming going on with these women and girls. Different organisations, international community, government. That’s why the community perspective change* (SP-IQ). These findings indicate that CRSV survivors in both contexts experienced stigma similar to what GBV survivors experienced around premarital sex, childbirth and a perceived loss of honour. However, community sensitization helped reduce blame. Some CRSV survivors in Nigeria still faced ostracization related to childbearing and could be stigmatised whether they returned with, or without a child. This provides important insight for service providers, to ensure sensitisation efforts are conducted for all GBV survivors.

### Theme 2: Stigma experienced by trafficked children and CBOW

Stigma experienced by trafficked children, (which could include child CRSV survivors), and by CBOW reportedly overlapped but with some clear differences.

### Abducted vs born in captivity

In both contexts, distinctions were made between abducted children and CBOW. Children who were abducted were almost always accepted back. In Iraq, while one Yezidi leader recognised community children had no choice in being trafficked, he also noted the community had no choice in accepting them back:

*It was difficult to handle them because they still had this ISIS thoughts … we welcome them, we have no problem with them, because it was not optional for them to go … But a long way is required to [reintegrate] them back [into society]. What should we do? We have no option* (YRL-IQ).

Comparatively, CBOW were reportedly less likely to be accepted in Iraq as noted by a service provider who explained the Yezidi community completely denied the children’s existence.

### Paternity

In both contexts, the paternity of CBOW was a key source of stigma, both because the father was part of the armed group and because the children were considered ‘fatherless’. In Nigeria, CBOW experienced paternity-related stigma, particularly linked to community perceptions and fear that the child would adopt the father’s violent behaviours. A caseworker shared community sentiments directed towards a case she was supporting: ‘*This one, he’s Boko Haram child.’ They said that the child has tendency to grow up like the father* (CWFGD-NI). This was similar in Iraq but was further linked to the child having a foreign/‘unknown’ father outside the Yezidi community and having a different religion (the father’s) to the mother. Several providers described notions around blood purity: *The Yezidi religion considers anyone who doesn’t come from Yezidi mother and father, as not a Yezidi, they have to have pure blood* (SP-IQ).

### Community-based stigma

In Iraq, CBOW were largely rejected by families and communities even before their return. While this in itself provides key insight, here we focus on the Nigerian context where high levels of stigma were reportedly directed towards CBOW. Caseworkers made a distinction between family and community stigma: *Most of the stigmatisation comes from the community, not from the immediate family … The [community] will stigmatise him and call him all sorts of names* (CWFGD-NI). These derogatory, often paternally linked names included, ‘son of Boko Haram’, ‘Eneji’ (child from the bush) and ‘Sheji (bastard) and reportedly led to child isolation and withdrawal. Further, stigma associated with ties to the armed groups were directed at all children (including child survivors), who were blamed for contributing to the destruction of their communities. One service provider explained that the community had witnessed the ‘*brutal slaughtering of people’* and blamed the armed groups for their displacement, making it difficult to accept or want anything to do with a child associated with them (SP-NI). An unintended source of stigma arose from the community perception that returnees were ‘untouchable’ and protected by government and community leaders. One service provider explained this tension was reinforced with providing reintegration kits only to returned children and not also to community children: *It’s just about, ‘hey this is somebody that destroyed my community, they come back, and you give them economic support’* (SP-NI).

### Changed behaviours

Other sources of stigma stemmed from perceptions about the activities returnees engaged in while in captivity, and changes in their attitudes, behaviours and practices particularly when they replicated them in the community. Behaviours eliciting aggression and talking about guns and violence were reportedly challenging for families to manage. A Nigerian service provider working in a rehabilitation centre noted that both returned boys and girls were ‘very aggressive’. Women emphasised how community avoided and feared girl returnees because: *… they train them how to shoot, how to place bombs. We are scared of them when they return … the girl grow[s] up in their hands. She will adopt whatever she is seeing or whatever they’re doing in the Bush* (WFGD-NI). They further explained that they would accept returnees with small babies because there was the opportunity to nurture and ‘train’ them at home, age acting as a potential protective factor. Themes of conversion, ‘indoctrination’ and ‘brainwashing’ were common across both contexts. In Nigeria, some believed that Boko Haram conducted incantations that involuntarily forced children to conduct severe acts even on their own communities. As such, in some cases, children were accepted back by the community based on this belief, enabling a protective factor, as explained by one service provider. In Iraq, the impacts of religious conversion and identity were seemingly more pronounced in comparison to the Nigerian context, with implications for reintegration. One service provider perceived that returned survivors were still suffering from ‘brainwashing’ and still ‘*praying, [and] fasting for Ramadan’* (SP-IQ). He experienced survivors feeling very upset and unable to accept when they were told they were Yezidi again. Another shared a similar sentiment about a CBOW who the community perceived to be brainwashed and converted: *[he’s] practicing Islam in the community … He was going for praying and calling and reading Quran and that was something that was not easy for [the family] to manage* (SP-IQ). Findings illustrate the different forms of stigma returned children faced, particularly at the community level and as a result of atypical and religiously converted behaviours. It particularly emphasises the differences between children who were trafficked and CBOW. One notable protective factor in Nigeria was the belief that abducted children committed atrocious acts based on voodoo, a belief which could facilitate their return. This was not an option for CBOW, who had very few protective factors (one being age) and often faced heightened risk, stigma and rejection, primarily rooted in beliefs surrounding their paternity.

### Theme 3: The experience of stigma by trafficked CRSV survivors

Survivors’ experiences of acceptance, rejection and stigma were acutely individual and could change over time. In both contexts, stigma and subsequent withdrawal and self-isolation was commonly reported. Yet, survivors also demonstrated the ability to navigate the wide-ranging consequences of stigma. In Nigeria, protective factors included relocating to new towns where they were unknown and being supported by their mothers and in Iraq, moving overseas and being supported by their husbands to claim the child was born prior to abduction. How survivors viewed themselves could also be a powerful protective factor, as one Yezidi survivor shared:

*My advice is that anyone who has this kind of experience they should never see themselves as something less or something weak in the community. They should see themselves as a very strong woman and they should be able to defend themselves and their rights and they should ask for their rights* (SV-IQ).

### Stigma from own family

Families’ acceptance of survivors and their children varied. In both contexts survivors were almost always *initially* welcomed back. Survivors in Nigeria and Iraq explained how they were received by their families: *we were received with both hands … both me and the baby [by mother]* (SV-NI); *Our family always treat us in a very special way, and they see us as a very special person. We get all the support needed from our family* (SV-IQ). However, Nigerian caseworkers explained that some survivors shared how their siblings were afraid of them and ran away and even after initial acceptance, underlying stigma related to their perceived association with the armed groups, the child’s paternity and behavioural changes could surface during family arguments. This stigma could persist decades post-conflict, as described by one participant’s experience in Sierra Leone: *It’s almost 18 years, but we still have those kinds of ways of stigmatising people back. ‘These guys were in the rebel line and this guy was born in the rebel line’* (SP-NI). Further, family reunification in Nigeria was more often an issue if survivors returned with a child. As one service provider explained: *A lot of family members accept them. It’s just when they come with children, that’s where the problem is* (SP-NI). Comparatively, in Iraq, CBOW were almost completely rejected by family and community, thereby rejecting survivors if they returned with CBOW. Government providers elaborated on cases they supported: *she managed to escape, but the family said we will not raise the child because it is Muslim, it is not Yezidi* (Govt-IQ).

### Families’ own experiences of stigma

Stigma experienced by family members from the community could eventually lead to survivors being rejected as described by a Nigerian government worker. However, one strong protective factor referred to by caseworkers in Nigeria, but not in Iraq, was the clear distinction in parents’ rejection. Survivors’ mothers most often accepted and supported their daughters and grandchildren regardless of how conceived, particularly compared to fathers who more frequently rejected and sent them away due to perceived and personal experiences of stigma. A service provider described one father’s response: *[The] father said ‘no you will not come to my house with this baby.’ He was willing to accept her, but he said ‘with this baby I will not accept you’* (SP-NI). Another described what happened when a mother accepted her daughter when the father was absent:

*‘Please bring back my daughter, I’m very happy to receive her.’ But the father was not around at the time and when he came, he was like ‘no, no, no no, no you have to take this child back I don’t care where you take her back to. If you want to kill her it’s your business’ …* (SP-NI).

Mothers reportedly understood that incidents of violence were not girls’ fault and could happen to anyone, even themselves. Caseworkers described supporting several women leaving unsupportive husbands to care for their daughters. They believed that father’s rejection often stemmed from shame they felt from the community based on: *‘your child is part of the persons that has destroyed this place, and we don’t want to see them’* (SP-NI), and that fathers were more interested in protecting themselves and not wanting to be identified as: *‘the father of a Boko Haram’* (ML-NI). Further, when girls faced parental rejection, their grandmothers and sisters were reported as other sources of support.

### Stigma from community

Community-based stigma was widespread and often led to serious consequences including preventing survivors from seeking essential services such as for sexual and reproductive health. In both contexts, fear of rejection of CBOW by the community led some survivors to either refuse rescue or escape or leave their children behind as reported by service providers and survivors:

*It was one of the worst feelings that I cannot describe because it’s caused by rape and it’s caused from someone from ISIS, so I was wondering how my community will see me, how can I face my community in this situation?. (SV-IQ)*

In Nigeria, service and government providers both emphasised: *family is not really the problem. Community is the problem* (SP-NI). However, community stigma could eventually lead to family rejection. Generally, community attitudes towards reintegrated fighters were mostly met with unease, mistrust, and anger. A Muslim leader explained: *We all know that they are not repentant* (MRL-NI). These views were seemingly extended to survivors by association of marriage, childbirth and community destruction. One service provider commented that some community saw returnees as ‘spies’, acting on behalf of the armed groups and further preventing incident reporting and access to services. Like their children, mothers often experienced name calling by the community like ‘*boko haram wives’*, ‘*Eh Sambisa’* and ‘*mother of the bastard’*. Yet, according to one government worker, unlike their mothers, children were not always considered an immediate threat. Other sources of stigma came from peers and friends as described by community girls: *Some of the friends might avoid her while some will not care … [They’ll avoid her] … thinking that maybe she doesn’t have a clean mind* (GFGD-NI). Service and government providers self-reflected on unintentional stigma from them: *It’s not easy. Even among we, the social workers, when you’re sensitising people, if you see a child coming from the Bush, definitely you have to look at that child twice* (Govt-NI). Another service provider explained a potential community-based protective factor that could facilitate survivors return. In certain insecure locations some communities believed having more people present increased their safety: *That community … for them they feel, ‘if we are more, it will be difficult for these guys to penetrate us’* (SP-NI).

In Iraq, despite broad acceptance of returnees (without CBOW), one survivor noted this did not include everyone in the community, perhaps revealing some underlying stigma: *I will not say everyone treated us in a very good way. But I can tell you majority of Yezidi community they were very welcoming* (SV-IQ). A survivor who returned with a CBOW did face rejection from her community as described by one provider who supported the case. Service providers stressed the Yezidi Religious Decree as an indispensable protective factor (not extended to CBOW), with one survivor validating this: *especially the religious leaders like Baba Sheikh. He really respect us. He welcomed us a lot.* (SV-IQ). Findings show that survivors often experience stigma at all levels of the social ecology, and that community-based stigma can be particularly harmful. Community-based stigma can contribute to triggering family rejection (later), by eliciting feelings of shame and loss of honour, felt particularly by fathers. How stigma and violence can intersect between mother and child was also revealed. Individual drivers of stigma but also shared risk was clear. Both faced stigma due to the associated links to the armed groups through marriage, birth, paternity and fear. Mothers could encounter stigma directed towards themselves, towards their child and as a result of the child’s paternity. Mothers also often faced higher rates of rejection when returning with a CBOW, with the mere fear of rejection preventing some from returning.

### Theme 4: Factors amplifying consequences of stigma and mother-child wellbeing

The broader environments survivors and CBOW returned to were reportedly very challenging across both contexts. Almost all participants spoke about the deteriorated socio-economic contexts emphasising the stress of limited livelihood opportunities, economic support and unavailable services. Other common issues mentioned were the impacts of displacement and community breakdown, high levels of GBV, including IPV and sexual exploitation and strong community patriarchal social norms, which held women and girls in traditional gendered roles. Survivors’ needs frequently outweighed the few available services, with cost, distance and awareness further limiting access. Service providers commonly spoke of reduced and short-term funding and significant gaps in services: *In general, the services is less than compared to the needs* (SP-IQ). Yet, survivors required the full range of basic services but also longer-term physical and mental health care, psychosocial support and ongoing reintegration support: *we need all the support [for] everything, not only psychological or financial* (SV-IQ). Further, while all children faced challenges with accessing limited services, service providers noted that CBOW faced some specific barriers including stigma and a lack of specialised services to meet their often-complex trauma needs. In Iraq, one particular legal barrier, was the inability for mothers to confer their own names to their children, having severe consequences for obtaining documentation, identity and citizenship for children and causing stress for mothers.

Moreover, many survivors were trafficked in childhood with some returning in adolescence. Caseworkers in Nigeria described that they saw different emotions surface between mother-child interactions and that while many wanted to stay with, keep and loved their children, others really struggled to parent, breastfeed and care for them, ultimately rejecting and maltreating them: *We have had instances where these teenage parents don’t even know how to breastfeed* (SP-NI). Community women shared a story of a returned two and a half year old boy who was being stigmatised by the community because of his behaviour, leading his mother to beat him: *Whenever he went out to play with other little children, he will be saying ‘I will shoot you’ to the other children. So, the mother kept beating him so, for now, the boy has changed* (WFGD-NI). This clearly illustrates how stigma can influence the mother-child relationship but also in some ways how this could be protective factor to prevent stigma against the child.

Across both contexts, a trend was revealed in how mothers treated children differently; those abducted with them versus CBOW. In Nigeria, service providers noted that they saw more concern shown for children born pre-abduction: *There’s neglect for the children conceived in the Bush. Rather than the children they had before the insurgency … They don’t give them food; they treat them badly* (SP-NI). Similarly, in Iraq a survivor explained that when she was abducted, she was already pregnant with her daughter, who she then gave birth to in captivity. She then gave birth to a boy and had two other unsuccesful pregnancies as a result of rape. On release, she took her daughter and handed over the boy: *I left him behind, I gave him to the armed group, and I left him and until this moment I know nothing about him. I have my daughter with me* (SV-IQ). Findings reveal how survivors’ experiences and feelings towards their children are uniquely individual. Emotions can be influenced by compounded socio-ecological stressors, including stigma, risk to further violence and limited services over time. These conditions, and unaddressed trauma from trafficking experiences may intensify ambivalence towards the child particularly when survivors face rejection and become sole caregivers. A shared factor around ‘fault’ crossed the findings involving where blame was placed: on GBV survivors compared to CRSV survivors and here, on CBOW compared to trafficked children. A clear issue emerging from our findings was that protective factors were available at only two socio-ecological levels: at societal level (service providers and faith leaders) and, selectively, at family level for some survivors (usually by female relatives). Protective factors were entirely absent at the community level and for CBOW. Gaps in protective factors indicate opportunities for intervention.

## Discussion

Findings show that trafficked CRSV survivors and their children can experience stigma at every socio-ecological level with severe consequences to their wellbeing, supporting existing literature on stigma. Findings also revealed how predictive factors of violence and CRVAWG could intersect between mother and child but that despite gaps in protective services survivors were still able to navigate stigma using resilience resources to cope with their situation and reduce risk. We contribute to the literature in two ways, through 1) understanding the interplay of stigma at the family and community levels, which is far less known, and how this can change over time, impacting mother-child relationship and risk factors to violence and, 2) contributing to comparative literature on two contexts that helps to emphasise potential protective factors for stakeholders to support. Notably, we also reveal how GBV survivors and trafficked CRSV survivors may be treated differently to the detriment of GBV survivors which has not been widely written about. We discuss, first, the need to address community experiences of conflict and the implications of deep-seated community norms that underly stigma. Second, we discuss the need to address family experiences of CRSV, given the severe consequences that community-based stigma can have on family experiences and rejection over time. Third, we discuss the need to address the intersecting stigma experienced by mother and child. Last, we expand on factors of ‘time’ and the importance of holistic services that meet the needs of all GBV survivors, including those who experience CRSV. Throughout, we outline effective strategies shown to counter stigma in other contexts.

### Community norms that underly stigma: addressing community experiences of conflict

In Nigeria and Iraq, all stakeholder groups confirmed that the most prevalent form of stigma was community-based. The fear of community-based stigma held so much power that it could reportedly prevent survivors escaping or accepting rescue from captivity with their children and returning to their communities. Where survivors did return, real and perceived stigma often led to isolation and withdrawal and prevented survivors from seeking services to manage complex experiences, delaying recovery. Our analysis of community-based stigma shows a combination of sources: existing social and gendered norms were violated (premarital sex and pregnancy, sexual violence, loss of purity, virginity and honour, paternity), and this experience was intensified by new conflict-related factors (CRSV, religious conversion, atypical behaviours). Two main factors explain community-based stigma dynamics: 1) blame related to the trauma, community destruction and loss of family witnessed during the conflict and the ongoing displacement and, 2) fear of the armed groups and ongoing insecurity. Community blame and fear were reportedly extended to survivors and CBOW who were perceived to be affiliated with the armed groups through marriage and paternity. As children have been shown to be ‘constant reminders’ to survivors such as in Bosnia [47], we suggest that returned mothers and CBOW can also act as reminders to the community of their conflict experiences. Notably, how communities choose or are influenced to see survivors, as heroines (Iraq) versus as part of the armed groups (Nigeria) can significantly influence survivors and their children’s reintegration experiences. In Nigeria, in populations attacked by Boko Haram, reintegration processes have largely failed to focus on community, which is critical for returnees’ reintegration [49]. Addressing community underlying social and gendered norms and community fear is therefore pivotal to mitigate stigma and support mothers and their children’s sustained reintegration.

To address community-based fear and stigma, it is essential to rebuild and restore community relationships and safety in culturally appropriate ways. Shevell and Denov [30] explain, *‘fostering resilience within communities* ***after a crisis*** *demands rebuilding, repairing and revitalising community strengths and resources in order to curtail individual vulnerability and augment well-being’*, where supporting sociocultural resources and networks are key. This rebuilding is premised on that conflict has ended. However, one of the challenges in Nigeria and Iraq is that both conflicts and insecurity have been protracted. On-going conflict continues to drive unemployment and limit educational opportunities. Reduced services impact unaddressed acute trauma and chronic stressors, all while returnees are seen receiving iatrogenic support [50, 51]. We suggest that these factors have helped to prolong and amplify community fear, stigma and animosity towards survivors and CBOW.

A few studies outline what has worked to address community-based stigma and acceptance. For example, research, covering multiple contexts shows positive results in improving mental health and reducing stigma for survivors when combining individual, household and community-based stepped-care programmes [36, 43, 52, 53]. Several collective activities which effectively counteract stigma exist. For example, community level exercises that correct false narratives and counter myths [52], and those that utilise cultural rituals to contextualise collective memory and help to reconcile different versions of events by faith leaders are some [54]. Significantly, in Uganda, economic agency has also been shown to reduce stigma by providing the opportunity to renegotiate status with family and community (Atim et al., 2018). Further, Koos [55] challenging feminist literature and the dominant idea that ‘*CRSV destroys the social fabric of communities’*, describes successful community-level actions in Sierra Leone, where survivors and their families took specific actions around social inclusion to counter ‘decay mechanisms’ (risk factors) including stigma, exclusion and discrimination. Notably, these counter mechanisms may not work for all CRSV survivors; for example, trafficked CRSV survivors with CBOW in Nigeria and Iraq, who relocated due to family or community rejection. For these survivors, their family and community social structures and wellbeing were impacted and broken. Other studies report that promoting successful reintegration by reducing community jealousy around targeted support of survivors [56]; building social cohesion, and reducing stigma through survivors’ civic engagement and contribution to their community (though found more successful for males) [8] are also successful measures. Our study contributes to showing the comparative influence Yezidi faith leaders held when they actively intervened in accepting survivors back, while sanctioning community stigma. Yet, this collective act of restoring community basic functioning was not extended to the collective acceptance of CBOW, thereby countering the acceptance of survivors who kept their children. This divergence between Iraq and Nigeria is important. It reveals both the positive impact of collective community acceptance of survivors, and the negative implications on mothers with CBOW, similarly reflecting children’s experiences like in Rwanda [30].

Our findings support research showing the criticality of providing holistic and whole community approaches [30, 57], across all levels to meet the needs and wellbeing of survivors, their children and their communities. We highlight that a careful balance is needed between individual and community needs. Individual-based trauma-rehabilitation programmes are vital for survivors, particularly those who are faced with community and family rejection and those having to manage deradicalisation and religious conversion. However, these interventions should also aim to bridge individual and community needs. A survivor-centred approach has been long advocated for by survivors and has been commonly applied by health and protection services in conflict settings. This approach has been essential for providers to re-empower survivors because it provides the space for individuals to make their own choices and decisions on how best to recover and be heard. However, a key part of this, is understanding that survivors’ wishes and choices may go against community norms, such as in Iraq, by keeping their children, resulting in implications for survivors such as rejection and relocation. Clark [57] challenges a survivor-centred approach and argues that by solely centring survivors’ needs this counteracts the functioning across social ecologies, marginalising and neglecting impacts of collective violence and how CRSV also impacts families and communities. Others note that interventions need to be cognisant of the dichotomy between individual needs and community needs, which may not align, thereby further marginalising survivors [54]. One method that could be explored to meet this need for balance is through collective healing and restorative justice mechanisms.

### Impacts of community-based stigma on family: addressing family experiences of CRSV

Not only does community-based stigma have implications for family members own wellbeing and coping but it can critically influence the relationship and support they provide to returnees. In Nigeria and Iraq, community-based stigma could influence family level rejection over time. In Nigeria, one of the clearest risk and protective factors was revealed by the difference between fathers’ and mothers’ support. As the closest layer surrounding the survivor, particularly during reintegration, Shevell and Denov [30] note that family resilience is: ‘*operating as an independent family unit, family members fulfil critical functions for one another including, belonging, identity and membership, economic support, socialisation, nurturance and protection*.’ At the same time, a predicative risk factor to violence against women and girls is having an absent or rejecting father [29]. For CBOW, not only did they have absent fathers, but their paternity was a direct source of stigma and rejection. In Nigeria, fathers often rejected their daughters and grandchildren, in fear of shame, lost honour and stigma. Comparatively, the value placed on motherhood acted as a strong resilience factor and is a key prospective area for intervention. In some places like Rwanda, this element of motherhood allowed mother’s to overcome stigma to care for and provide for their children [30]. Our findings extend this value on motherhood by revealing how survivors’ mothers, grandmothers and sisters were strong support systems. This was to the extent that in Nigeria, some grandmothers left their own husbands to support their daughters and grandchildren, despite social sanctions and limited economic support, an area for further research.

### Impacts of intersectional stigma: addressing mother-child experiences of stigma

Mothers’ navigation and displays of parenting were revealed in a few ways. First, according to some participants, some mothers, to protect their children from community stigma, beat them when they displayed atypical and aggressive behaviours; this emphasises the importance of providing positive parenting interventions, while addressing stigma. Some survivors also abandoned children, leading to further marginalisation and denial of social benefits similarly shown elsewhere (Amnesty, 2018). Others returned to the armed groups to be with their children, effectively contributing to community disruption and social disintegration, and further complicating women’s interactions with their children, family and community [58]. Abducted community children also faced community ostracization due to their changed behaviours, religious conversion and witnessing violent acts and use of weapons. Compared to CBOW, they were welcomed back into their communities, underscoring the dichotomy and strong social norms around paternity and community identity. Some mothers, in circumstances where they had children conceived prior to abduction, chose not to keep CBOW. Where they did, treatment often negatively differed and placed children at risk of further harm. A study in Uganda similarly showed that when CBOW misbehaved, they reported receiving harsher punishments than other children in the home [59].

Parenting programmes represent a positive approach to promote CBOW well-being and community cohesion. Intergenerational resilience [30] was clear in survivors’ care for their children and their own mother’s care for them and their grandchildren. Positive parenting practices (and education/livelihood) particularly when survivors were rejected and became the sole support resource for their children were essential but rarely available. As stated by several participants, girls often become mothers in childhood or adolescence without the support, resources and skills to parent. When women and girls experience sexual violence, they can experience a range of consequences [4]. When they face family and community rejection and become sole caregivers, they and their children can face additional gendered barriers to livelihoods and risks including to sexual exploitation. These layered circumstances can further interlink complex psychological traumas and wellbeing between mother and child [18]. A study based in Bosnia also found positive impacts on mother’s mental health and their children’s weight gain and psychosocial functioning [60]. Particularly critical, given the funding gaps in aid, the intervention involved an inexpensive psychosocial programme that involved weekly meetings over a period of time that promoted children’s wellbeing through parental support and education and included a mother-child component that focused on the children’s development, trauma and social interaction [60].

### Factors of ‘time’ and holistic GBV services

In both contexts, the factor of time played an important variable on stigma in three ways. First, ‘time’ influenced survivors and their families’ experiences of (underlying) stigma. This could change (initial) family acceptance to (later) rejection as a result of a number of factors including community-based stigma. Second, ‘time’ influenced CBOW, particularly if they were babies on return as they were not seen as immediate threats unlike older children and their mothers. However, the relentlessness of stigma and its intergenerational impacts should not be underestimated which continues to be prevalent in contexts like Rwanda, Bosnia, Sierra Leone, and Liberia 30 years post conflict. In particular, CBOW in these contexts continue to experience barriers to rights and services, the lack of recognition by governments and face questions around identity and paternity as they age [12, 22]. Third, where survivors chose or were coerced to leave their children behind, prolonged time apart combined with stigma and daily stressors, reportedly resulted in poor mental health and in some cases return to the armed groups. This ‘time’ factor emphasises intersecting risk factors of violence over time and over generations, giving insight to areas for intervention. Further, GBV experienced post-conflict can be an extension of violence experienced pre-conflict, while the conflict can create new risks [21]. GBV incidents reportedly increased in both contexts and new drivers formed, emphasising the importance for providers to deliver holistic services for survivors experiencing any form of GBV. Complementary services to address specific needs for CRSV can then be provided, such as addressing conflict-related stigma, legal deficiencies and facilitating documents and birth registration for CBOW. It fundamentally shows the importance of addressing unequal social and gendered norms that condone VAWG, perpetuate victim blaming and put all women and girls at risk of GBV before, during and after conflict.

### Limitations

A few notable limitations. First, uneven data collection occurred across the two contexts. More data was collected in Nigeria, as one site visit and one country visit to Iraq was cancelled due to insecurity. This imbalance influenced cross-context comparisons and led to overrepresentation of some stakeholder groups. Factoring this, findings are first framed by what was shared across both sites, followed by Nigerian data and then differences or similarities observed in Iraq. Second, we relied on third-party information of CBOW experiences, thereby excluding their perspectives. Third, sample bias may have occurred. Fewer survivor interviews were held than planned and only those who accessed services were interviewed therefore underrepresenting those who did not access services. Data collection with male community members was unfeasible due to insecurity and difficulties in aligning facilitator gender so their perspectives are also not reflected. Last, interpretation by caseworkers may have potentially influenced data.

## Conclusion

Formerly trafficked CRSV survivors and their CBOW can experience individual and joint forms of complex interrelated and severe stigma and violence at all levels of society. The overall objective of this paper was to explore how stigma intersects between mother-child and how survivors navigate and cope with stigma towards themselves and their children in Nigeria and Iraq. Findings show that numerous interrelated risk factors to violence and stigma were present across all social ecologies but that survivors utilise several resilience resources to cope. **Protective factors** that help survivors navigate and cope with stigma, included sensitisation and awareness by service providers; relocation to a new town or asylum overseas; and supportive husbands who accept CBOW as their own - an area for further exploration. Most notably, in Nigeria, motherhood and support from grandmothers was a strong protective factor. A vital protective factor for returned Yezidi survivors in Iraq was the Yezidi religious decree. This authoritative ruling created a new norm that sanctioned victim blaming and effectively countered community animosity. This was not extended to CBOW putting them and their mothers at risk. **Risk factors** that undermine survivors’ ability to cope with stigma included perceived violations of existing social and gendered norms (premarital sex/childbirth, sexual violence, loss of honour) and new conflict-related forms of stigma and violence (CRSV, paternity, religious conversion, association to armed groups). Paternity, particularly for CBOW can be a key source of stigma, distress and rejection for children and their mothers. Gaps in services and barriers to essential services can also compound experiences and delay recovery and reintegration. Other risk factors were returning with a child, iatrogenic aid, changed and aggressive behaviours and community anger, animosity and fear towards the armed groups and by association to returnees. Importantly, community-based stigma and fear could undermine family acceptance over time and sustained reintegration in both contexts. On-going insecurity helped to prolong blame, stigma and feelings of animosity towards returnees, acting as a barrier to reintegration, social cohesion and peacebuilding and preventing restoring positive relationships with community and returnees. Notably, while community sensitisation was a protective element that at the surface level suppressed stigma in the short term, it did not address underlying root causes that impact all survivors. This study reveals some protective factors stakeholders can strengthen to promote resilience and some risk factors to tackle. Addressing these factors can contribute to WPS, YPS and meeting SGDs 3 (good health and well-being), 4 (quality education), 5 (gender equality), 16 (peace, justice and strong institutions) and 17 (partnerships and goals). For donors and policymakers, the importance of funding and ensuring system-strengthening approaches that tackle short- and long-term unequal norms that drive stigmatisations for all survivors was clear.

## Electronic supplementary material

Below is the link to the electronic supplementary material.

Supplementary Material 1

## Data Availability

The qualitative dataset supporting the conclusion of this article is available on request.

## Acronyms

CBOW: Children born of war
CRSV: Conflict related sexual violence
CRVAWG: Conflict-related violence against women and girls
CWFGD: Caseworker FGD
FGD: Focus group discussion
GBV: Gender-based violence
GFGD: Girl’s FGD
GOVT: Government
I/NGOs: International/non-governmental organisations
IDI: In-depth interview
IQ: Iraq
ML: Male leader
MRL: Muslim Religious Leader
NI: Nigeria
SEM: Social ecological model
SP: Service provider
SV: CRSV survivor
SVRP: Sexual violence-related pregnancy
UN: United Nations
WFGD: Women’s FGD
WL: Woman leader
YRL: Yezidi Religious Leader
