# Supplementary material for: Social ecosystems of trafficked conflict-related sexual violence survivors and their children: navigating and coping with intersecting stigma and violence in Nigeria and Iraq
Source: Int J Equity Health. 2026 Mar 31;25:127. doi: 10.1186/s12939-026-02828-9 (PMC13169662; doi:10.1186/s12939-026-02828-9)
Supplement: Supplementary file 1 — Supplementary Material 1 [file 12939_2026_2828_MOESM1_ESM.docx]

**IDI GUIDE #1: WOMEN**

**Distress Protocol**

If at any point during the interview, the participant becomes distressed, acknowledge her feelings and experience. Offer her the opportunity to pause or stop the discussion and or psychosocial support if required.

Checklist

1. Introductions
2. Explanations of the research
3. Objective of the IDI
4. Consent procedures

(2.) A serious consequence of conflict related sexual violence (CRSV) for women and girls in many places in the world is childbirth resulting from rape. This research is exploring the exploring the needs of mothers and their experiences around available, accessible and acceptable services and how they navigate/negotiate several challenges especially around stigma.

(3.) The objective of this interview is to understand your experience during conflict and your immediate, inter-mediate and long terms needs for service support. [**If appropriate**: *It is also trying to understand the situation of your child and the support they need and the experiences of stigma you may have faced*].

We would appreciate hearing about your situation and how you managed to cope but also your recommendations to other survivors and to service providers.

(4.) Obtain informed consent.

| **#1 WOMEN IDI** | | | |
| --- | --- | --- | --- |
| 1 | If you are comfortable, can you share with us your marital status, education level and age? |  |  |
|  | Can you tell me about the situation/challenges of women and girls in this community   - 1. Before the conflict \| During the conflict \| After/now the conflict |  |  |
| 2 | Can you share your experience and what happened to you during the conflict? | Q1 |  |
| 3 | What were your immediate needs after your exposure to violence? | Q1, Q4 |  |
| 4 | Who helped you? / Who did you go *to* for help? | Q1, 3,4 |  |
| 5 | What services did you use? |  |  |
| 6 | Can you tell me about the birth (of your child?) and what happened. | Q1, Q2 |  |
| 7 | What happened to the child after delivery?  FACILIATOR NOTE: *Depending on response (*death, left with someone, abandoned*) DO NOT ASK any question related to a child including questions 8, 12b/c. | Q2, Q1 |  |
| 8 | What were the immediate needs of your child?   1. What support did you need to take care of your child? 2. What are the needs of your child now? 3. How did you manage the relationship with your other children? | Q2 |  |
| 9 | How did your family react to your situation? | Q3 |  |
| 10 | How did your community react to your situation? | Q3 |  |
| 11 | How did religious leaders and/or faith actors react to your situation? |  |  |
| 12 | Have you experienced any challenges in obtaining legal documentation and rights for yourself and/or your child?   1. Can you explain what these were/are? 2. How did you decide on a name for the child? *(first and last)*   *NOTE: Context specific* only if it is normal that ethnicity and/or religion is recorded in legal documents, ask the following question.*   1. Were you able to choose the ethnic group and/or religion of the child if the perpetrator is likely from a different group? | Q4, Q2 |  |
| 13 | If you met another woman/girl that experienced a similar situation to you, what advice would you give to her? |  |  |
| 14 | Is there anything you would like to share or questions you would like to ask? |  |  |

Thank you for participating in this interview.

Facilitators note:

1. Explain what will happen with this information.
2. Provide referrals information.
3. Provide blank copy of informed consent.

**IDI GUIDES: #2 Community/faith leaders | #3 Government | #4 Service providers**

(1.) Checklist

1. Introductions
2. Explanations of the research
3. Objective of the IDI
4. Consent procedures

(2.) This research is exploring the experience and needs of women and girls who experience conflict-related sexual violence (CRSV) during conflict. A serious consequence of CRSV for women and girls is childbirth resulting from rape. We are trying to understand survivors and their children’s situation and their experience in accessing services.

(3.) Share objective of research.

| **COMMUNITY/FAITH LEADERS** |
| --- |
| The objective of this interview is to understand how the community responds to women and girls’ exposure to conflict-violence and how the community supports them in the immediate, inter-mediate and long-term. It is also trying to understand the situation of any child they may have had and the support/services they need. ***As a leader in this community***, we would appreciate hearing your experience when it comes to this subject, how the community feels about this topic and your recommendations. |
| **GOVERNMENT** |
| The objective of the government stakeholder/institution interviews is to understand the nature and extent of the response to this population. ***As an official*** who works in a key department responding to the needs of women and children, we would appreciate hearing about your department’s initiatives and policies on what is in place (in terms of services) to respond to this populations needs. |
| **SERVICE PROVIDERS** |
| The objective of this interview is to understand the nature and extent of available, accessible and acceptable services and recommendations including resources being used (or suggested) to 1) respond to the needs of mothers and their children in humanitarian settings 2) discuss the barriers and challenges of mothers to AAAQ services and 3) discuss the challenges in delivering AAAQ services. ***As an expert***, who works in the field of GBV/child protection, we would appreciate hearing about your understanding of their situation and strategies on how to better respond to this vulnerable population. |

(3.) Obtain informed consent.

(4.) Questions.

| **#2 COMMUNITY/FAITH LEADERS IDI** | | |
| --- | --- | --- |
| 1 | Can you tell me about the situation/challenges of women and girls in this community   1. Before the conflict \| During the conflict \| After/now the conflict |  |
| 2 | What are the strategies in place to protect and keep children safe and healthy in your community?   1. Are there situations where children are no longer protected? | Q2 |
| 3 | Now, I’d like to focus on conflict-related sexual violence perpetrated by people outside this community that specifically affects women and girls. What types of violence against women and girls are most common during conflict?   1. How is rape defined in this community? | Q3 |
| 4 | What happens to a woman/girl when she is exposed to CRSV?   1. Where would she go to for help? What support would she need? | Q1, 3,  4 |
| 5 | How would her family/community respond to her situation? |  |
| 6 | How would community/religious leaders support her?   1. Would it be different for a women and girl? 2. Are there specific religious ceremonies to support the woman? | Q1, 4 |
| 6 | If a woman became pregnant after she was raped during conflict by someone **not** from the same community, what would community leaders/ religious leaders tell her?   1. **FAITH LEADERS:** Are there traditional or cultural structures that support survivors that are exposed to CRSV and may become pregnant? | Q3, 4 |
| 7 | If a child was born from CRSV, what would happen to the child?   1. Are there strategies are in place to protect these children? 2. How would the woman’s family support the woman/child? 3. Would there be a difference between the woman’s male family members and her female family members? 4. What would the community say about her situation and the child? 5. How would the child be treated by family/ community/ community leaders? 6. Are there specific religious ceremonies to support the child? | Q1, 2, 3 |
| 8 | What are the community/faith leader’s responsibility supporting and (re)integrating women back into the community? What about the children? |  |
| 9 | **FAITH LEADERS ONLY:** Are there religious text/teachings that;   1. support survivors who are exposed to CRSV and become pregnant from a perpetrator outside the same community? 2. talk about the protection of children who have been born from sexual violence during conflict? | Q1, 2, 3, 4 |
| 9 | What types of services (*health, legal, safety/security, religious, cultural*) are available to survivors of CRSV in this community?   1. Who are providing these resources? 2. Are there any barriers to accessing these resources and what are they? 3. Do you know of any legal policies in place that support these women/children? 4. **FAITH LEADERS**: Are any of these resources managed by the religious institution? | Q3, 4 |
| 10 | Is there anything you would like to share or questions you would like to ask? |  |

| **#3 GOVERNMENT INSTITUTIONS IDI** | | |
| --- | --- | --- |
| 1 | Can you tell me about the situation/challenges of women and girls in this community?   1. Before the conflict \| During the conflict \| After/now the conflict 2. What types of VAWG are most common during conflict? |  |
| 2 | Does the government/this institution have any specific strategies in place to support women who have experienced CRSV and as a result become pregnant? Children? | Q1, 2, 4 |
| 3 | What support services does this institution have in place *(health, legal, safety/security, religious, cultural)* for when a woman/girl is exposed to CRSV/SVRP? | Q1, 3, 4 |
| 4 | What about after the woman has delivered the child? | Q1, 2, 3 |
| 5 | What are the policies/laws related to women’s rights and safety in this situation? | Q4 |
| 6 | What are the policies/laws related to the children/ and children born from foreign/unknown fathers? Citizenship? Documentation?  ***NOTE: Context specific* if it is normal that ethnicity and/or religion is recorded in legal documents, ask the following question.***   1. Are women allowed to freely choose the ethnic group and/or religion of the child if the perpetrator is likely from a different group? 2. Are women allowed to freely decide on a name for the child? (first/last). | Q3, 4 |
| 7 | Is there anything you would like to share or questions you would like to ask? |  |

| **#4 SERVICE PROVIDERS IDI** | | | |
| --- | --- | --- | --- |
| 1 | Can you briefly describe the context you are working in and the nature of the programmes and services you currently provide/support? |  |  |
| 2 | Are you aware of any programmes/strategies/services that have been effective in the response to CSRV survivors or CBOW (including your own or others) in humanitarian settings?   1. What programmes/services you are providing? 2. What tools/strategies you are using? 3. What made them effective/successful? 4. What were the key challenges and lessons learned? 5. How were these programmes designed/adapted to the context? | Q4 |  |
| 3 | Can you describe the situation of CRSV survivors in the contexts you work? | Q1, 3 |  |
| 4 | Can you describe the situation of CBOW in the contexts you work? | Q2, 3 |  |
| 5 | In what way does the knowledge, attitudes and behaviours of frontline service providers influence access to services? | Q3, 4 |  |
| 6 | Can you explain the procedure of what happens when a women or girl who has been exposed to CRSV or a sexual violence pregnancy?   1. Do you have any guidelines for responding and can you explain what these are. 2. Do any referral pathways exist for survivors of sexual violence? If so, what are they? Do they usually get referred to you? If so, by who? Do you refer women and girls to other services? 3. Do they have challenges in accessing services? Why, why not?   **COUNTRY SPECIFIC QUESTIONS**   1. **IQ:** What were the alternative care arrangements of children who were left behind in Syria after delivery? 2. **NI:** Do you have any information regarding children left behind with Boko Haram or the children who were released by Boko Haram. How do the community perceive them? | referral  info |  |
| 7 | How do you think stigma or discrimination against CRSV survivors /CBOW influences their lives?   1. What impacts does it have on the mother/child bond? 2. What impacts does it have on reintegration? | All |  |
| 8 | What do you think is the best way to approach the issue of CRSV survivors / CBOW on a 1) individual basis 2) community basis;   1. What do you think some of the key challenges would be? 2. Which family and community members need to be engaged and what strategies have been effective in doing this? 3. What is your experience of engaging religious and community leaders on CRSV survivors/CBOW? Are there traditional and cultural ways of healing/managing the situation of CRSV survivors/CBOW? Challenges? | Q1, 2, 3 |  |
| 9 | What are the political/legal frameworks for addressing VAWG at the national and local level that support or hinder the rights of survivors/CBOW?   1. Do you know of any specific laws related to conflict-related sexual violence? 2. How do you think the state laws and policy frameworks influence the lives of CRSV survivors/CBOW? Positively? Negatively? 3. How do you think the state laws and policies positively or negatively influence the provision of services and support to CRSV survivors and CBOW? 4. How do these affect the CBOW rights and access to services, citizenship and documentation? | Q4 – esp. legal |  |
| 10 | Are there any gaps in existing resources you think need to be addressed?   1. What would you suggest including in a guidance/service package targeting the needs of CRSV survivors/CBOW? 2. How would you monitor and evaluate the effectiveness of your programme? 3. Who would you suggest coordinating with and what methods would you recommend? | Q4, 3 |  |
| 11 | What would you recommend including in addressing the needs of CRSV/SVRP survivors and CBOW?  What would you specifically suggest including in a guidance document to support GBV/CP case workers in strengthening services for women, girls and children in this situation? | All |  |
| 12 | Is there anything you would like to share or questions you would like to ask? |  |  |

Thank you for participating in this interview.

Facilitators note:

1. Explain what will happen with this information.
2. Provide referrals information.
3. Provide blank copy of informed consent.

**#5 FGD GUIDE - FEMALE/MALE COMMUNITY MEMBERS**

This guide will be used for participatory focus group discussions with community groups.

**Important note:** If any participant approaches any of the facilitators and discloses personal accounts of violence or requests additional information, provide them with the contact information of the nearest GBV programme staff or other GBV and health service providers in the area. Referral information will be available to all researchers and translators.

Checklist

1. Introductions
2. Explanations of the research
3. Objective of the PFG
4. Consent procedures

(2.) A serious consequence of conflict related sexual violence (CRSV) for women and girls is childbirth resulting from rape. This research is exploring the experience and needs of women who experience sexual violence related pregnancies, the experiences of their children and what support (services) they need and how they navigate/negotiate several challenges.

(3.) The objective of this participatory focus group discussion is to help us fill in a hypothetical story with made up characters.

(FOR FACILIATOR ONLY: To determine community reactions to violence against women and girls, and specifically conflict related sexual violence pregnancy, help seeking behaviour from a community perspective, stigma associated with sexual violence related pregnancies and children and knowledge of services.)

As members of this community, we appreciate your time and hearing about the situation of women and girls in your community and if these stories happen in your community, what will happen.

(4.) Obtain informed (verbal) consent. (Must be recorded in lieu of signed consent).

**FREE LISTING EXERCISE**

**Objective:** to create a list of violence against women and girls that exists in the relevant community and explore the levels and types of responses, allowing a broader introduction discussion regarding violence the community has been exposed to.

**FACILITATOR INSTRUCTIONS**

1. Place five premade different coloured large sticky notes titled ‘home’, ‘school’, ‘work’, ‘community’, ‘conflict’ on a wall/board. (In English or the local language). Also prepare three large separate sticky notes with ‘before conflict’, ‘during conflict’, ‘after conflict’.
   1. If one type of violence occurs in multiple places, re-write the type of violence on a sticky note and place it under both places.
2. The note taker should be taking separate notes of any discussion that takes place while the facilitator is writing on the sticky notes and facilitating the discussion. **Take a photograph of the board.**
3. Once the list is finalised, the facilitator will divide the sticky notes into three separate columns of when the violence was most prevalent – **before conflict, during conflict, after conflict**.
4. After this session, a **photograph must be taken of the board** for data analysis purposes.

| **FREE LISTING EXERCISE INSTRUCTIONS**   1. We would like to know about all the types of violence that women and girls are exposed to this community. We will write the types of violence on these sticky notes. You can help us to understand where the type of violence occurs – home, school, work, community or during conflict. If a type of violence happens in two or three different places, we will place it under each heading ‘place’.    1. What are some of the types of violence that you know of or that you have heard that exist in this community?    2. What about types of violence that happens during conflict? *(if no more response and no one mentions CRSV-SVRP – probe)* 2. There are many different ‘times’ where violence can occur. We would now like your help to understand under each of these headings, if there was a time that each time of violence occurred more i.e., before conflict started, during conflict, or after conflict (now). |
| --- |

**VIGNETTE – OPEN ENDED STORY**

**Objective:** to better understand community reactions to violence against women and girls, and specifically conflict related sexual violence pregnancy, help seeking behaviour from a community perspective, stigma associated with sexual violence related pregnancies and children and knowledge of services.

**FACILITATOR INSTRUCTIONS**

1. The ideal size for a PFGD is 8-10.
   - Where possible, participants should be broadly considered in separate groups as;

- Female community members
- Male community members

1. Conduct separate sessions with women and men.
2. The PFGD should be facilitated in the local language.
3. The PFGD must be conducted in a private space that is free from distractions.
4. The discussion will be based around a short open-ended story (vignette). It will involve fictional characters who must make decisions about their situation.
5. Each story will be contextualised to each local context for example through names of characters. *No significant changes to the storyline should be made.*
6. One or more facilitators should conduct the discussion and at least one facilitator/notetaker should take notes. Template provided which should include recording the *date, location, time started/time ended, facilitator name/note taker name and participant summary (female/male).*

| **VIGNETTE EXERCISE INSTRUCTIONS**   1. Facilitators introduce themselves to the group. 2. Facilitator obtains verbal consent from the group by reading out loud in the local language. **Audio record verbal consent if possible. Explain the use of the recorder.** *“We need to keep a record that you have agreed to participate. We will use this recorder to do so. If you all agree that the discussion can be recorded, we will continue to use it. If not, we will only record that you agree and thereafter turn it off.”* 3. Ask the participants to introduce themselves. Remind the participants that their names will not be recorded by the notetaker (***and that the recorder can be turned off during this part***). 4. Explain the session; *“I am going to read to the group the beginning of a fictional story. We would like you to help us to finish the stories of what would happen to the characters if they were in your community. We will ask some specific questions to help us discuss as a group.”* 5. Read the following stories, followed by the discussion questions. |
| --- |

**STORY**

**Read the following to the relevant group:**

*I am going to read the beginning of a story about a woman in a community like yours. I want your help in filling in her story as if she were in your community. I will ask you some questions after I read the story.*

(*Samira*) is 25 years old. (*Samira*) is married with two children. (*Samira*) had to flee (*name of town*) with her husband and children due to conflict in the area they lived. While they were fleeing (*Samira*) was abducted with several other women. During her abduction, (*Samira*) became pregnant.

*(Samira)* is 19 years old. *(Samira)* used to enjoy school with her friends in her village. During a violent attack on her village *(Samira)* was kidnapped/abducted together with many other girls. While in captivity, *(Samira)* became pregnant. *(Samira)* managed to escape while she was carrying her baby boy.

**Facilitator:** We are now going to ask you some questions about the story. Before we start, does anyone have any questions about the story? (*Only clarify details in the story and do not accept other responses (yet*).

***Facilitators note:*** *Remind the group the story is not based on any particular person and that there is no ‘correct’ answer.*

**VIGNETTE QUESTIONS**

**Samira focused:**

1. Who (if anyone) will Samira talk about what happened to her?
   1. Will she tell anyone in her family?
   2. What about anyone else in her community?
   3. What about religious leaders?
2. What do you think the responses of the people she told would be?
   1. Were they helpful?
3. Will Samira try to go anywhere to get help?
   1. **Will she try to access any services? What are they?**
4. What will happen to Samira when she gets to the services?
   1. What will the doctors/nurses/police officers/counsellors do? How will they respond?
   2. Will she be satisfied with these services and how she is treated?
5. Why wouldn’t Samira go to (*family, religious, community leaders, health care providers, counsellors, legal centre, other appropriate options)?*
   1. Would Samira know about the options available?
   2. Why or why not?
6. What do you think will happen to Samira?
   1. What will happen to her health?
   2. What will happen to her marriage?
   3. What about her children?
7. Where should Samira have gone?
   1. What service do you think she could have received if she could go there and why is it important for her to receive that type of service?

**Child focused:**

1. What would happen to Samira when she gave birth?
2. What would happen to the child Samira gave birth to?
   1. Would Samira want to keep the child? Why, why not?
3. Would Samira have the choice of what to do or will someone else advise her on what to do? Who would Samira go to for advice?
   1. What would Samira’s husband advise her to do?
   2. What would Samira’s family advise her to do?
   3. What would Samira’s friends advise her to do?
   4. What would religious leaders advise Samira to do?
   5. Of all the people that Samira might go to for help, whose opinions would be most influential.
4. If Samira chose to keep the child, what would the community say?
   1. How would the community support her or the child?
5. **Now if Samira was a 14-year-old girl what would be different?**
   1. Who would she tell?
   2. Where would she go for help?
   3. What would her family tell her to do?
   4. What would the religious leaders advise her to do?
